# Case Report: Hydroxychloroquine-induced generalized pustular psoriasis in a pediatric patient with systemic lupus erythematosus

**DOI:** 10.3389/fped.2025.1652376

**Published:** 2025-10-13

**Authors:** Xue Wang, Linlin Dong, Hui Qiu, Yihuai Xu, Linxiaoyu Kong, Weiran Zhou

**Affiliations:** ^1^ Department of Pediatric Nephrology and Rheumatism and Immunology, Children’s Hospital Affiliated to Shandong University, Jinan, Shandong, China; ^2^ Department of Pediatric Nephrology and Rheumatism and Immunology, Jinan Children’s Hospital, Jinan, Shandong, China

**Keywords:** generalized pustular psoriasis, hydroxychloroquine, systemic lupus erythematosus, pediatric, adverse drug reaction

## Abstract

Hydroxychloroquine (HCQ) therapy is the main treatment for systemic lupus erythematosus (SLE); however, rare adverse effects, including generalized pustular psoriasis (GPP), have been predominantly reported in adults. We herein report the first case of GPP caused by HCQ in a pediatric SLE patient. A 7-year-old girl with SLE developed fever, hepatic dysfunction, and disseminated pustules 3 weeks after starting HCQ. Histopathological examination revealed the characteristic features of GPP, including epidermal hyperkeratosis with parakeratosis, pustule formation above the stratum spinosum and acanthosis. Neutrophils and lymphocytes were observed in the superficial and mid-dermal vascular plexuses. HCQ therapy was discontinued, and the patient received methylprednisolone, intravenous immunoglobulin, meropenem, and hepatoprotective therapy. After 9 days of treatment, the pustules had largely resolved and inflammatory markers had returned to normal. This unprecedented pediatric observation underscores HCQ as a potential trigger for GPP in pediatric patients with SLE and highlights the importance of immediate HCQ discontinuation for optimal outcome. Interleukin-17 and interleukin-36 cytokine pathways may synergistically contribute to pathogenesis, suggesting a role for targeted therapies.

## Introduction

Psoriasis is a chronic inflammatory disease which can be divided into non-pustular and pustular forms. Among non-pustular psoriases, plaque psoriasis (also known as psoriasis vulgaris) is the most common subtype, accounting for approximately 90% of cases (1, 2). The chronic inflammatory response characteristic of this condition is primarily driven by the presence of pathogenic T cells in the skin. The T helper 17 (Th17)-interleukin17 (IL-17)-interleukin23 (IL-23) axis constitutes the central mechanism underlying disease pathogenesis (1). Psoriasis cum pustulatione denotes the fleeting, scattered, sterile micropustules that arise within or at the margins of pre-existing psoriatic plaques and patients are usually afebrile with no systemic inflammatory signs. Pustular psoriasis (PP) comprises generalized pustular psoriasis (GPP), impetigo herpetiformis and localized pustular psoriasis. Whether palmoplantar pustulosis should be classified as localized pustular psoriasis or regarded as a distinct entity remains controversial (1, 2).

GPP is a rare, yet potentially life-threatening autoinflammatory dermatosis. GPP presents with erythema, pain, fever, and systemic manifestations; within hours, pinpoint pustules emerge, coalesce, and form lakes of pus (1, 2). IL-17 and interleukin-36(IL-36) are key drivers in the pathogenesis of GPP. The pro-inflammatory activity of IL-36 is further amplified by feedback regulation within the IL-17/IL-36 axis (3). It exhibits heterogeneous progression patterns, ranging from self-limiting to chronic refractory presentations. GPP most commonly is idiopathic and not triggered by drugs. Hydroxychloroquine (HCQ) therapy, the main treatment for systemic lupus erythematosus (SLE), is generally well-tolerated but has been increasingly recognized as a potential trigger for psoriatic flares, especially pustular variants. However, reported cases of HCQ-associated GPP have only been in adults.

Here, we present the first case of HCQ-induced GPP in a pediatric patient with SLE, expanding the spectrum of HCQ-related adverse effects and linking the immunopathogenesis of these conditions. This case underscores the need for careful monitoring to detect early this rare but serious adverse drug reaction in pediatric patients. It also highlights the need for further research to optimize treatments for this population with dual autoinflammatory pathologies.

## Case report

A 7-year-old girl presented with a history of a recurrent malar erythema for 3 months, bilateral thigh myalgia for 3 weeks, and febrile illness for 2 days. Physical examination revealed classic cutaneous manifestations, including a butterfly-shaped facial erythema and palmar erythema, accompanied by arthritis. Laboratory investigations demonstrated hematologic (leukocytopenia: 3.62 × 10^9^/L) and immunologic abnormalities [hypocomplementemia: C3 0.223 g/L, C4 <0.0575 g/L; positive ANA (1:1000) and anti-dsDNA antibodies]. These findings confirmed a diagnosis of SLE. Initial management comprised high-dose methylprednisolone pulse therapy (500 mg/day for 3 days) followed by oral prednisone (55 mg/day), supplemented with mycophenolate mofetil (0.25 g BID), belimumab (240 mg/dose), and HCQ (75 mg BID).

**Figure 1:**
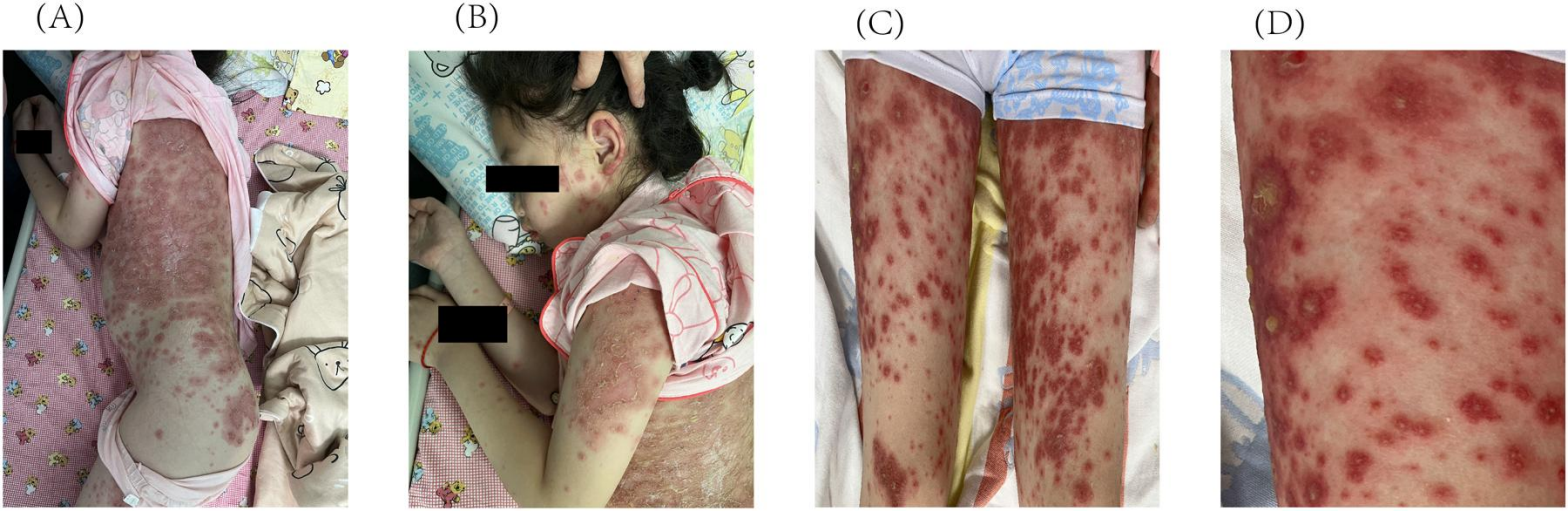
Initial cutaneous manifestations of pustular psoriasis. Multiple pustules on the intense erythema are observed on **(A)** the trunk, **(B)** the face and upper limbs, **(C)** the lower limbs. **(D)** Enlarged image of the lower limbs.

**Figure 2:**
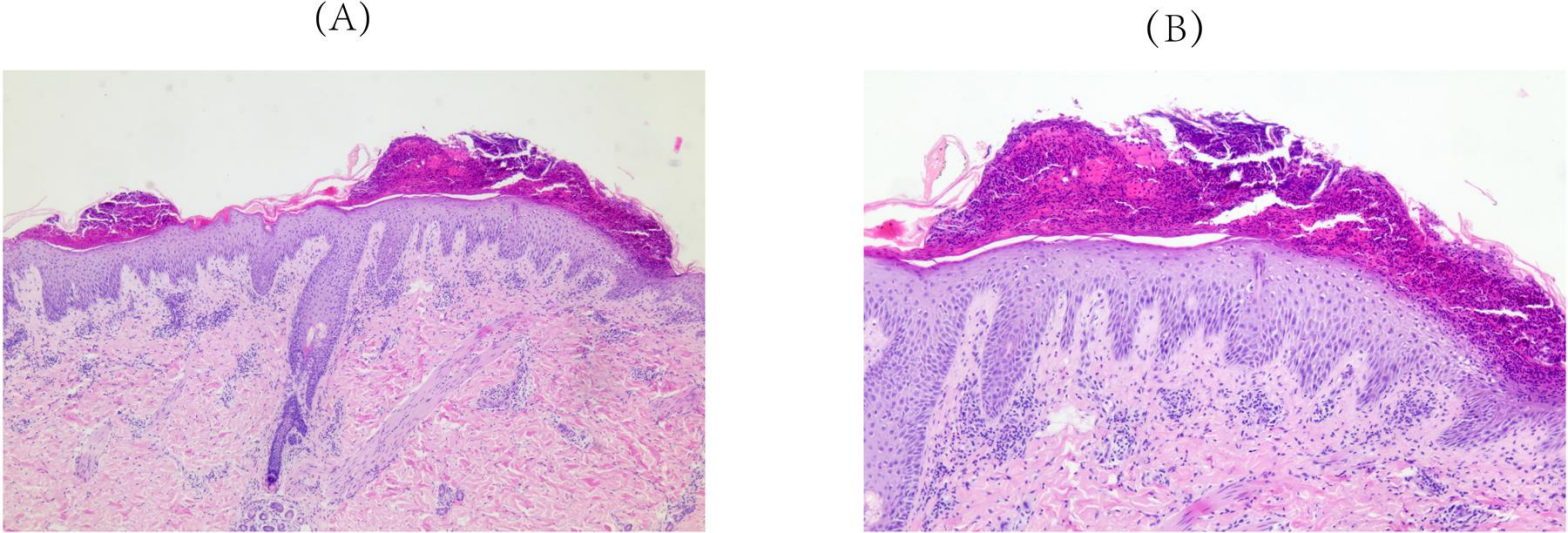
Pathological findings of skin biopsy (right upper limb). Epidermal hyperkeratosis with parakeratosis, pustule formation above the stratum spinosum and acanthosis. Neutrophils and lymphocytes localizes to the superficial and mid-dermal vascular plexuses were observed. **(A)** Original magnification ×50, **(B)** Original magnification ×100; hematoxylineosin staining.

Approximately 3 weeks after HCQ therapy, the patient developed a progressively worsening pruritic pustules, which progressed from the lumbar area to the facial, truncal, and extremity regions (Figure 1). A skin biopsy of the right upper limb pustular eruptions showed epidermal hyperkeratosis with parakeratosis, pustule formation above the stratum spinosum and acanthosis. Neutrophils and lymphocytes were observed in the superficial and mid-dermal vascular plexuses (Figure 2).

**Figure 3:**
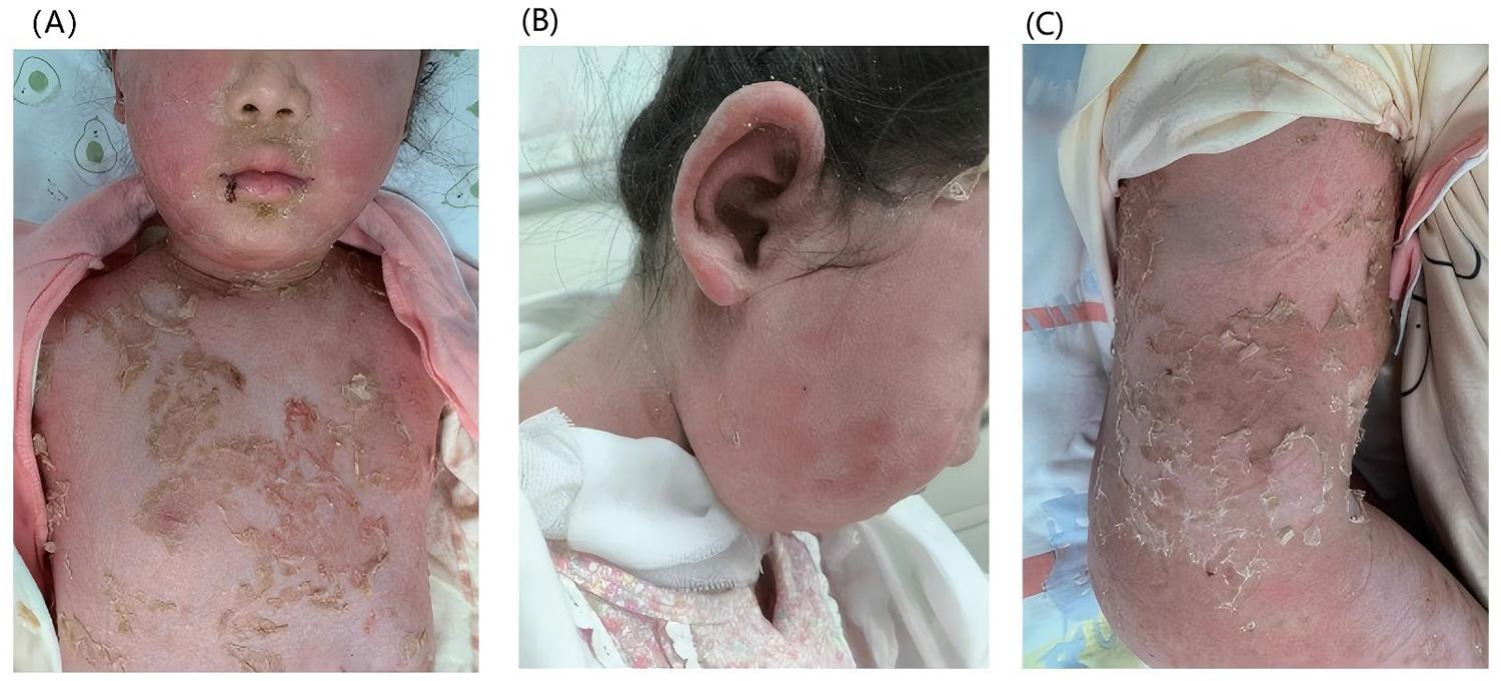
Cutaneous manifestations after nine days of treatment. After treatment, the pustules gradually subsided with subsequent desquamation: **(A)** the trunk, **(B)** the face, **(C)** the trunk and the lower limbs.

The clinical course was complicated by pyrexia with substantially elevated acute phase reactants (C-reactive protein: 93.59 mg/L; procalcitonin: 0.62 ng/ml), necessitating sequential antimicrobial therapy, initially with azithromycin followed by meropenem, and adjunctive intravenous immunoglobulin administration. Significant clinical improvement was observed with therapeutic intervention comprising intravenous administration of methylprednisolone (22 mg BID), immunoglobulin, ammonium glycyrrhizinate-cysteine complex, and vitamin C; oral medications (mycophenolate mofetil 0.25 g BID); immediate cessation of HCQ; and localized topical therapy (including silicone oil cream and compound miconazole nitrate cream). After 9 days of therapy, the pustules had largely resolved with desquamation, demonstrating marked clinical improvement (Figure 3). Although secukinumab biologic therapy was recommended during treatment, it was postponed based on parental preference. At the 12-month follow-up, the patient maintained complete remission without skin lesion recurrence. The prednisone dose was successfully tapered to a maintenance dose of 7.5 mg/day with sustained disease control.

## Discussion

GPP is a rare variant of psoriasis, accounting for approximately 1% of cases with varying global incidence (4). Some adult-onset GPP typically occurs in patients with prior psoriasis history, while pediatric-onset GPP frequently develops in the absence of prior psoriasis history. It differs between adults and children, with pediatric cases often being less severe. However, severe cases can be life-threatening and often exhibit recurrent episodes (5).

GPP demonstrates histopathological hallmarks of psoriasiform hyperplasia and spongiform pustules of Kogoj, with minimal or absent eosinophils. In contrast, acute generalized exanthematous pustulosis (AGEP), histopathology reveals sub corneal/intraepidermal pustules amid spongiosis, a neutrophil-rich infiltrate with eosinophils. Focal keratinocyte necrosis and dermal edema may be present (6, 7). Unlike our pediatric SLE patient, Liccioli et al. (8) reported the first pediatric case of AGEP induced by HCQ treated for Sjögren Syndrome, posing such a drug as a possible trigger also in children. Additionally, an Iranian study (9) analyzing 20 female patients with HCQ-induced pustular eruptions—whose clinical and histopathologic features did not fully meet criteria for AGEP or PP—identified overlapping characteristics of both conditions. The authors first proposed that this HCQ-triggered overlap presentation may represent a new entity, while acknowledging that further studies are required for validation. Pustular drug reaction with eosinophilia and systemic symptoms (pustular DRESS) evolves from maculopapular eruptions to folliculocentric pustules, accompanied by facial edema, lymphadenopathy, eosinophilia, and multiorgan involvement (notably hepatotoxicity). Histopathology shows dense superficial and deep perivascular infiltrates rich in lymphocytes, eosinophils, and atypical lymphocytes, often with interface dermatitis (10, 11). In this pediatric case, with pustules appearing approximately three weeks after medication use. The histopathology demonstrated classic features of pustular psoriasis. No eosinophils, necrotic keratinocytes were observed. These findings support a diagnosis of GPP and are inconsistent with AGEP. Furthermore, the absence of facial edema, eosinophilia, or multi-organ involvement—along with the histopathological features—rules out pustular DRESS.

Triggers of GPP include abrupt medication withdrawal including corticosteroids or other anti-psoriatic medications, bacterial/viral infections, taking antimalarial agents, immunization, pregnancy, psychological stress, and hypothyroidism (12). Several studies have demonstrated that the most common trigger of GPP is the withdrawal of systemic glucocorticoids (13–15). Conversely, GPP flares have also been reported in patients with non-pustular or localized pustular psoriasis after glucocorticoid exposure (16). The most commonly reported case is the development of GPP in patients with plaque psoriasis treated with corticosteroids (17). In the present case, the child had no prior history of psoriasis and was still in the initial, full-dose induction phase of glucocorticoid therapy. The GPP improved without discontinuing the glucocorticoids, thereby excluding glucocorticoid as the trigger of GPP in this patient. Additionally, the onset of GPP was not preceded by infection at any site, indicating the absence of an infectious trigger. The elevation of acute-phase reactants on day 5 after admission is considered to reflect a systemic inflammatory response. Nonetheless, secondary bacterial infection cannot be completely excluded.

HCQ has an excellent safety profile, dermatological manifestations, particularly maculopapule, erythema, urticaria and pruritus, represent the predominant skin-related adverse reactions, affecting up to 10% of treated patients. Long-term administration may lead to pigmentation changes in skin and mucous membranes. Notably, severe cutaneous adverse reactions including GPP, DRESS, and AGEP remain exceedingly uncommon (18, 19). Recent studies show it may worsen psoriasis and induce GPP, GPP of pregnancy, and palmoplantar pustulosis (20–22). Ten cases of HCQ-induced GPP in adult patients have been documented (Table 1) (20, 21, 23–29). Notably, five of them had SLE as the underlying condition. Currently, although psoriasis and SLE share common pathogenic mechanisms, their coexistence remains relatively rare. Tselios et al. reported 63 cases of psoriasis among 1,823 SLE patients in Canada (incidence rate 3%) (30). According to a report by Ali et al., the incidence of GPP is low, with an estimated prevalence of concurrent GPP and SLE at 0.69% (31). Additionally, Yu et al. reported a case of GPP coexisting with subacute cutaneous lupus erythematosus (32).We herein present the first documented case of HCQ-induced GPP in a pediatric patient with SLE. The causality assessment was carried out using Naranjo scale (Table 2), yielding a total score of 8. This score indicates a “probable” causal relationship between HCQ and the adverse reaction. Despite the potential for rechallenge to raise the score by 2 points (to a “definite” level), ethical and clinical concerns rendered it inadvisable. No other potential GPP triggers, as previously enumerated, were identified in this child. Importantly, upon further inquiry regarding the medication history, the parents explicitly reported that the child developed dry skin and pruritus following the initiation of oral HCQ. With cumulative exposure to the drug, pustular eruptions emerged. After discontinuing HCQ, the patient continued the original treatment for the underlying disease and received ongoing symptomatic supportive care. The condition was relieved, further confirming the role of HCQ in causing GPP.

**Table 1:** Cases of generalized pustular psoriasis caused by hydroxychloroquine.

References	Sex	Age (years)	Disease^b^	Duration of the HCQ treatment^a^	Dosage of HCQ (mg/day)	Treatment after HCQ withdrawal	Outcome
Friedman SJ (23)	Female	60	Rheumatoid arthritis	3 weeks	400	None	Improved
Vine JE (24)	Female	38	arthritis	3 weeks	200	Glucocorticoid, methotrexate	Improved
Gravani A (25)	Female	40	Psoriasis	1 month	200	Glucocorticoid, cyclosporine	Improved
	Female	37	Psoriasis	3 weeks	100	Glucocorticoid	Improved
Maglie R (26)	Female	70	Mixed connective tissue disorder	2 weeks	Not described	Glucocorticoid	Improved
Shindo E (20)	Female	34	SLE	3 weeks	200	Glucocorticoid granulocyte and monocyte, Adsorption apheresis cyclosporine	Improved
Ryoo YW (21)	Female GPPP	34	SLE	3 weeks	200	Glucocorticoid, cyclosporine	Improved
Kishibe M (27)	Female	64	SLE and psoriatic arthritis	4 weeks	300	Apremilast ixeKizumab Brodalumab	Improved
Akaji K (28)	Female	38	SLE	Not described	Not described	Secukinumab	Improved
Minami Y (29)	Female	55	SLE	16 days	200	Glucocorticoid, cyclosporine Spesolimab	Improved
Our case	Female	7	SLE	3 weeks	150	Glucocorticoid Immunoglobulin Mycophenolate Mofetil	Improved

^a^ Duration of the HCQ treatment: The interval between hydroxychloroquine (HCQ) administration and the onset of pustular eruptions.

^b^ Disease: Underlying diseases before hydroxychloroquine administration.

**Table 2:** Causality assessment of suspected ADR using naranjo scale.

Question	Yes	No	Don't know/NA	Reasons for rating/Score^a^
Are there previous conclusive reports on this reaction?	+1	0	0	There are previous conclusive reports on this reaction (Table 1). +1
Did the adverse event appear after the suspected drug was administered?	+2	−1	0	The adverse event appeared after the suspected drug was administered. +2
Did the adverse reaction improve when the drug was discontinued or a specific antagonist was administered?	+1	0	0	The adverse reaction improved when the drug was discontinued. +1
Did the adverse event reappear when the drug was re-administered?	+2	−1	0	The drug was not re-administered. +0
Are there alternative causes (other than the drug) that could on their own have caused the reaction?	−1	+2	0	No other causes were identified that could have contributed to the reaction. +2
Did the reaction reappear when a placebo was given?	−1	+1	0	No placebos were used. +0
Was the drug detected in blood (or other fuids) in concentrations known to be toxic?	+1	0	0	No. +0
Was the reaction more severe when the dose was increased or less severe when the dose was decreased?	+1	0	0	The patient developed dry skin and pruritus shortly after initiating low-dose hydroxychloroquine therapy. GPP onset post-dose increase; resolved after stopping drug. +1
Did the patient have a similar reaction to the same or similar drugs in any previous exposure?	+1	0	0	No. +0
Was the adverse event confirmed by any objective evidence?	+1	0	0	Photographs of changes in the **pustular eruptions** before and after discontinuation of medication served as objective evidence. +1
Total score				8

^a^ Score: Definite:≥9, Probable:5–8, Possible: 1–4, Doubtful: ≤0.

Emerging evidence has implicated HCQ as a precipitating factor for GPP through the inhibition of epidermal transglutaminase, leading to keratinocyte hyperproliferation via IL-17 overproduction (33). Elevated IL-17 levels in both GPP and SLE suggest a shared pathogenesis (34), with IL-17 inhibitors demonstrating promise for SLE treatment (35). Secukinumab has shown efficacy in adult refractory cases, including lupus nephritis with comorbid psoriasis (36) and HCQ-induced GPP with concurrent SLE, demonstrating rapid improvement and sustained remission (27, 28). Pediatric GPP respond similarly to secukinumab (37). Moreover, IL-36 can bind to the IL-36 receptor, initiating an inflammatory cascade that promotes the expression of various cytokines and neutrophil-attracting chemokines. The expression of IL-36 can be enhanced by inflammatory cytokines derived from other immune cells (such as CD4^+^ T cells), including IL-17A and IL-23. Increased levels of IL-36 may further drive the differentiation of IL-17-producing CD4^+^ T cells. These cytokines could establish a positive-feedback loop through autocrine and paracrine mechanisms (38, 39). Interleukin 36RN may be the most common causative gene for GPP. Genetic testing was indeed performed for this child. The results identified a heterozygous missense variant in the IL36RN gene: NM_012275.3: c.129C>A (p.Ser43Arg). But this variant was interpreted as one of “Uncertain Significance” (VUS) according to the ACMG guidelines. The IL-36 receptor inhibitor, spesolimab, achieves remission in adults with refractory GPP/SLE cases after conventional therapy failure (29), with preliminary pediatric data on GPP showing favorable outcomes (40). These findings suggest that inhibitors of the IL-17 and IL-36 pathways are promising therapeutic options for this complex clinical scenario.

GPP is a rare yet severe dermatological condition that can be life-threatening. Although HCQ has been implicated as a potential trigger of GPP, its use requires careful risk-benefit evaluation. For HCQ-induced GPP in pediatric patients with SLE, the prognosis largely depends on timely diagnosis and appropriate therapeutic intervention. While biological agents have been well-documented in the literature as effective treatment options for idiopathic GPP, our case report suggests that prompt drug discontinuation may obviate the need for biologic therapy in cases of drug-induced GPP. Nevertheless, owing to its rare incidence, existing evidence remains limited. Hence, We still need further validation of clinical data.

## Patient perspective

The parents of this patient expressed satisfaction with both the treatment process and the clinical outcome; however, they declined the proposed therapy with secukinumab.

## Data Availability

The original contributions presented in the study are included in the article/Supplementary Material, further inquiries can be directed to the corresponding author.
